# Acute Coronary Syndrome in Elderly Patients: How to Tackle Them?

**DOI:** 10.3390/jcm13195935

**Published:** 2024-10-05

**Authors:** Fabiana Lucà, Felicita Andreotti, Carmelo Massimiliano Rao, Giuseppe Pelaggi, Mariacarmela Nucara, Carlo Ammendolea, Laura Pezzi, Nadia Ingianni, Adriano Murrone, Donatella Del Sindaco, Maddalena Lettino, Giovanna Geraci, Carmine Riccio, Claudio Bilato, Furio Colivicchi, Massimo Grimaldi, Fabrizio Oliva, Michele Massimo Gulizia, Iris Parrini

**Affiliations:** 1 Cardiology Department, Grande Ospedale Metropolitano di Reggio Calabria, 89100 Reggio Calabria, Italy; fabiana.luca92@gmail.com (F.L.); giuseppepelaggi@hotmail.it (G.P.); maricanucara@gmail.com (M.N.); 2 Cardiology Department, A. Gemelli, University Hospital, IRCCS, 00100 Roma, Italy; felicita.andreotti@unicatt.it; 3 Cardiology Department San Martino Hospital, 32100 Belluno, Italy; carlo.ammendolea@ulss.belluno.it; 4 Cardiology Department, Ospedale Civile dello Spirito Santo, 65100 Pescara, Italy; laurape@live.it; 5 ASP Trapani Cardiologist Marsala Castelvetrano Districts, 91022 Castelvetrano, Italy; nadiaing@hotmail.it; 6 Cardiology Unit, Città di Castello Hospital, 06012 Città di Castello, Italy; 7 Cardiology Unit, Nuovo Regina Margherita Hospital, 00100 Rome, Italy; donatelladelsindaco@virgilio.it; 8 Cardiology Unit, IRCCS San Gerardo dei Tintori Hospital, San Gerardo, 20900 Monza, Italy; maddalena.lettino@irccs-sangerardo.it; 9 Cardiology Department, Sant’Antonio Abate Hospital, ASP Trapani, 91100 Erice, Italy; giovannageraci@hotmail.com; 10 Cardiovascular Department, Sant’Anna e San Sebastiano Hospital, 95122 Caserta, Italy; carminericcio8@gmail.com; 11 Department of Cardiology, West Vicenza Hospitals, Arzignano, 36100 Vicenza, Italy; claudio.bilato@aulss8.veneto.it; 12 Clinical and Rehabilitation Cardiology Department, San Filippo Neri Hospital, ASL Roma 1, 00100 Roma, Italy; furio.colivicchi@gmail.com; 13 Cardiology Department, F. Miulli Hospital, Acquaviva delle Fonti, 70021 Bari, Italy; m.grimaldi@miulli.it; 14 Cardiology Unit, ASST Grande Ospedale Metropolitano Niguarda, 20100 Milano, Italy; fabri.oliva@gmail.com; 15 Cardiology Department, Garibaldi Nesima Hospital, 95122 Catania, Italy; michele.gulizia60@gmail.com; 16 Cardiology Department, Mauriziano Hospital, 10128 Torino, Italy; irisparrini@libero.it

**Keywords:** elderly, acute coronary syndrome, ACS, non-ST-segment elevation myocardial infarction, NSTEMI, ST-segment elevation myocardial infarction, STEMI, percutaneous coronary intervention, PCI

## Abstract

Elderly patients diagnosed with acute coronary syndromes (ACS) represent a growing demographic population. These patients typically present more comorbidities and experience poorer outcomes compared to younger patients. Furthermore, they are less frequently subjected to revascularization procedures and are less likely to receive evidence-based medications in both the short and long-term periods. Assessing frailty is crucial in elderly patients with ACS because it can influence management decisions, as well as risk stratification and prognosis. Indeed, treatment decisions should consider geriatric syndromes, frailty, polypharmacy, sarcopenia, nutritional deficits, prevalence of comorbidities, thrombotic risk, and, at the same time, an increased risk of bleeding. Rigorous clinical assessments, clear revascularization criteria, and tailored approaches to antithrombotic therapy are essential for guiding personalized treatment decisions in these individuals. Assessing frailty helps healthcare providers identify patients who may benefit from targeted interventions to improve their outcomes and quality of life. Elderly individuals who experience ACS remain significantly underrepresented and understudied in randomized controlled trials. For this reason, the occurrence of ACS in the elderly continues to be a particularly complex issue in clinical practice, and one that clinicians increasingly have to address, given the general ageing of populations. This review aims to address the complex aspects of elderly patients with ACS to help clinicians make therapeutic decisions when faced with such situations.

## 1. Introduction

Cardiovascular diseases (CVD) are highly prevalent among older individuals [1]. The incidence of CVD has been estimated to rise with age, reaching approximately 40%, 75%, and 86% among patients aged 40–59, 60–79 years, and ≥80 years, respectively [2].

Notably, the incidence of acute coronary syndromes (ACS) among elderly patients is expected to rise due to the population’s increasing age [3].

Advanced age has been identified as an independent prognostic factor for adverse outcomes in patients with ACS [4], strongly correlating with complications following a revascularization procedure [4]. Indeed, elderly patients experience higher in-hospital mortality compared to younger cohorts, as well as increased rates of CV events and mortality at one year FU post-percutaneous coronary intervention (PCI) [5].

Nonetheless, elderly patients are underrepresented in randomized controlled trials (RCTs) [6]. This is also evident in clinical practice, where this kind of patient is often excluded from invasive treatment strategies, remaining a neglected category.

Indeed, elderly patients often present with greater complexity due to frailty, multiple comorbidities, and various geriatric syndromes [7].

However, according to the latest guidelines [8], an invasive approach should be considered for all patients experiencing ACS, as advanced age alone is not an exclusion criterion.

Conversely, a comprehensive risk assessment is strongly advisable to choose the most appropriate strategy [2].

Consequently, there is an urgent need to assess frailty in patients with ACS by performing a multidimensional evaluation, including a comprehensive Geriatric Assessment (CGA), before invasive management.

However, the decision-making process regarding the balance between risks and benefits remains complex in clinical practice, and the elderly population is often undertreated.

The aim of this paper is to address this complex issue, providing physicians with a practical tool.

## 2. Epidemiology

The risk of coronary artery diseases (CAD) in early adulthood progresses steadily over time [9]. Advanced age stands out as a major risk factor for ACS and independently predicts higher mortality rates. Non-ST-segment elevation myocardial infarction (NSTEMI) is the most common form of ACS in elderly individuals and is characterized by elevated mortality rates. Approximately 60% to 65% of ST-segment elevation myocardial infarction (STEMI) occurs in individuals aged 65 and older, with 28% to 33% occurring in those aged 75 and older. Moreover, a substantial 80% of all myocardial-infarction-related deaths occur in individuals aged 65 and above [10].

## 3. Pathophysiology

Age-related cardiac changes may lead to increased left-ventricular (LV) wall thickness, alterations in diastolic filling patterns, impaired LV ejection, and heart rhythm disorders [11].

Consequently, there is a heightened prevalence of LV hypertrophy, heart failure (HF), atrial fibrillation (AF), and CAD among older people [12,13,14,15,16].

**Figure 1:**
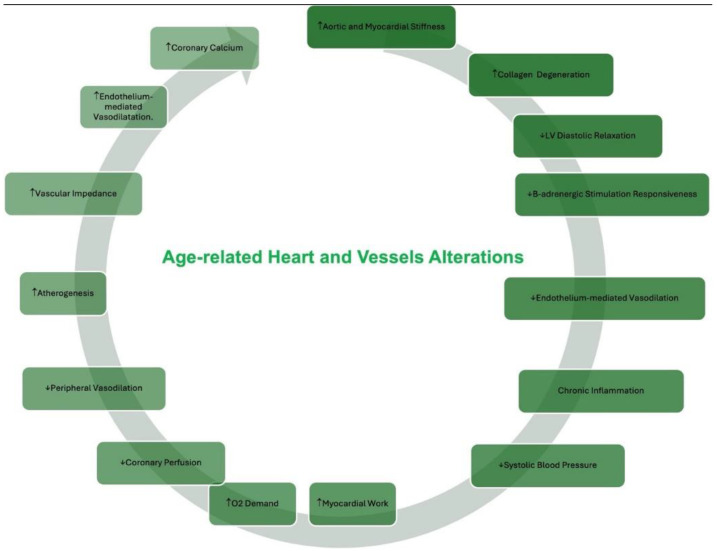
Age-Related Cardiovascular Changes. ↑: increase of, ↓ reduction of.

The pathophysiological mechanisms of CAD align closely with those observed in old age due to common underlying mechanisms, including fibrosis, inflammation, increased arterial and ventricular stiffness, elevated oxidative stress, endothelial dysfunction, reduced nitric oxide production, and impaired ability to increase coronary blood flow in response to heightened metabolic demands [17] (Figure 1). Furthermore, regardless of concurrent comorbidities and additional risk factors, CAD is intricately linked to various changes that significantly increase the risk of ACS in elderly patients [2]. Additionally, advancing age is associated with greater complexity in coronary anatomy. Elderly individuals frequently present with more severe conditions, such as triple vessel disease (TVD) and left main coronary artery disease (LMCAD), often compounded by calcific lesions [2].

## 4. Comorbidities

The prevalence of comorbidities increases significantly with age, with approximately 70% of adults aged > 75 years with multiple active chronic conditions. Risk stratification must account for these conditions [18]. Arterial hypertension (AH), chronic kidney disease (CKD), AF, and chronic obstructive pulmonary disease (COPD), either alone or in combination, have been considered the most frequent chronic disorders observed in the elderly. Importantly, comorbidities often coexist with polypharmacy, increasing the risk of drug interaction and complications [19,20]. Anemia is a prevalent condition among the elderly that deteriorates the quality of life and decreases life expectancy. It can trigger ACS or exacerbate chronic conditions [21].

Another important condition that affects the decision-making process is dementia. The stress of acute events may contribute to deteriorating cognitive function. In these patients, an increased risk of delirium has been reported, leading to more prolonged hospitalizations.

### 4.1. Clinical Manifestations

Elderly patients presenting with ACS often exhibit atypical symptoms rather than typical chest pain, referred to as ‘anginal-equivalent’ symptoms, such as dyspnea, syncope, and malaise. Additionally, their electrocardiograms (ECGs) may demonstrate non-diagnostic findings, including newly developed conditions such as AF, LVH, or bundle branch block (BBB), which can hinder timely recognition of ACS. Elevated troponin levels are frequently observed in the elderly due to factors such as replacement fibrosis and renal dysfunction, necessitating careful interpretation in the clinical context. Furthermore, elderly ACS patients commonly present with multiple concurrent conditions, such as CKD, electrolyte disturbances, and anemia, complicating both medical treatment and interventional procedures. When concomitant severe aortic stenosis (AS) occurs, symptoms can range from unstable angina to severe hemodynamic compromise. These manifestations may mimic acute myocardial infarction (AMI) and can be confusing in the clinical diagnosis [22].

STEMI often manifest with unusual symptoms in older adults, which can lead to increased mortality rates and mechanical complications in this population [23]. The clinical characteristics of STEMI in elderly patients differ significantly from those in younger cohorts [23]. It has been estimated that 20% of STEMI patients aged over 85 are admitted with diagnoses unrelated to ACS, whereas this is true for less than 5% of STEMI patients under 65 [24]. Furthermore, the elderly frequently have pre-existing ECG abnormalities and present with atypical clinical manifestations, making the detection of new ECG changes more challenging [24].

A comprehensive understanding of the diverse presentations of ACS in older adults is essential for ensuring prompt and effective diagnosis and treatment. Diagnosing ACS in this demographic may be delayed due to the presence of atypical symptoms like dyspnea, syncope, and confusion, which are often non-specific and more pronounced in women. Non-specific ECG changes, such as AF, LVH, or BBB, are also frequently encountered in older adults, contributing to delays in ACS.

### 4.2. MINOCA (Myocardial Infarction with Non-Obstructive Coronary Arteries) in Elderly

MINOCA encompasses conditions such as plaque rupture/erosion, thrombosis, embolization, spontaneous coronary artery dissection (SCAD), epicardial or microvascular spasm, and demand–supply mismatch [25]. These cases are more prevalent in females. Women with MINOCA tend to be older and more frequently hypertensive compared to men, who are more likely to be smokers, have a higher BMI, and a history of prior ischemic cardiac events [26,27]. Although troponin levels are comparable between sexes, women have a higher GRACE score and worse renal function [28,29,30]. SCAD is more common in women, while non-obstructive coronary artery disease is more frequent in men [27,28,29,30]. At presentation, men are more likely to have STEMI, whereas women present with a higher Killip class [31]. At discharge, men are more likely to receive dual antiplatelet therapy and statins [32]. Among patients with MINOCA, women exhibit higher mortality rates compared to men, although mortality rates are similar between sexes in MIOCA (Myocardial Infarction with Obstructive Coronary Artery Disease). Over a 28-month follow-up, major adverse events (MAEs) are more common in women than in men in both groups. With advancing age, the prognosis for women around 55 years with MINOCA worsens, showing a delay of approximately 15 years compared to MIOCA. Women under 70 years with MINOCA experience worse outcomes compared to older women with MINOCA, and special attention should be given to women with MINOCA under 70 years of age [33]. The delay in disease onset in women compared to men, reaching similar levels around menopause, is likely due to the waning protective effects of estrogen over 10–15 years [27]. This may contribute to a predominance of SCAD or microcirculatory issues in women [34]. Intravascular imaging or functional microcirculatory testing should be considered to identify mechanisms unrelated to plaque, as these have distinct therapeutic and prognostic implications [33].

### 4.3. Invasive versus Conservative Strategy in Elderly

Elderly patients are less likely to receive interventional treatment compared to younger patients despite the higher rates of in-hospital mortality [35].

However, due to the advancements in revascularization procedures and in antithrombotic strategies, PCI adoption has also increased in recent decades in the elderly, with a notable reduction in intra-hospital complications and mortality [36].

It has been well-established that there are no substantial differences in STEMI management between older and younger patients [37,38].

Indeed, even among the elderly, primary PCI represents the first-line strategy for STEMI, with confirmed safety and efficacy [37,38]. Importantly, in elderly patients diagnosed with STEMI, an invasive approach has been shown to be superior to fibrinolysis [39].

However, STEMI-related factors, such as delay of detection, ECG alterations, systolic dysfunction, clinical stability, and patient-related factors, such as the burden of comorbidity, severe cognitive failure, and low life expectancy, should be carefully considered [37,38].

**Figure 2:**
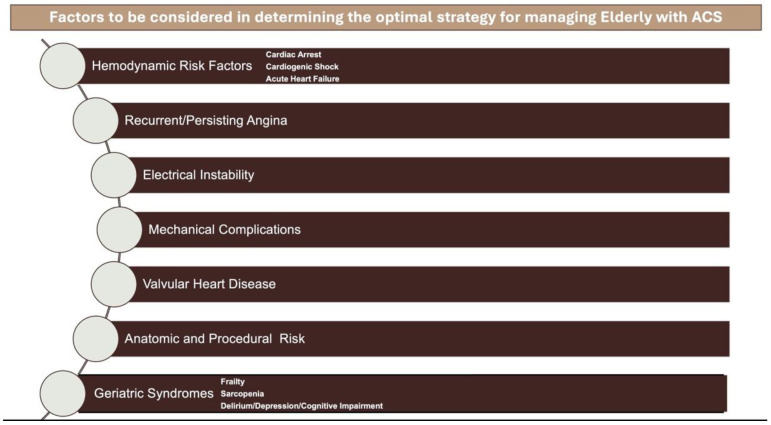
When determining the treatment strategy for ACS in elderly patients, it is essential to consider functional and cognitive capacity, quality of life, comorbidities, risk of complications, hemodynamic status, electrical and clinical stability and assessing anatomic and procedural risk. Taking these factors into account helps to personalize the treatment approach. Abb: ACS; Acute Coronary Syndrome; VHD: valvular heart disease.

Conversely, when exploring the NSTEMI-ACS in the elderly, the issue becomes slightly more complex and the evidence becomes less clear, requiring a comprehensive risk evaluation based on CV and non-CV elements [37,38] (Figure 2).

Therefore, a risk stratification of patients with NSTEMI should be performed to determine the appropriate management strategy and establish whether the patient is at low or high risk. Risk assessment scores, such as the TIMI and the GRACE risk score, should be utilized.

A less use of invasive cardiac procedures in older patients has been reported, despite their elevated risk factors, such as HF, diabetes mellitus (DM), and higher GRACE scores. According to the GRACE registry, accounting for 16% of octogenarians, the very elderly patients who underwent revascularization experienced lower rates of HF, recurrent ischemia, major bleeding, and death compared to those who received only medical therapy [40]. Revascularization proved highly beneficial in reducing the primary combined endpoint of stroke at six months, death, and AMI across all age groups, including the young, old, and very old, with a notable reduction in mortality at six months. Importantly, these benefits were achieved without a corresponding increase in the risk of stroke.

From data of a meta-analysis of three studies comparing a routine invasive versus a selective approach (FRISC II, ICTUS, and RITA-3) on 5467 NSTEMI patients, (51.3%, 33.3%, and 15.3% aged ≤ 65, ≤74, ≥75 years, respectively), a routine invasive strategy resulted in a long-term reduction in the composite endpoint of CV death and AMI in the elderly [41].

In the FRISC II trial, on 2457 patients with a mean age of 66 years, a lower AMI incidence and a non-statistically significant reduction in mortality at 6 months have been reported in patients invasively treated [42], with a more significant beneficial effect at 5-year follow-up in terms of mortality reduction in elderly patients [43].

In the RITA-3 trial, involving 1810 patients with an average age of 62 years, at four months, a reduction in refractory angina has been observed in those undergoing the invasive strategy [44].

In the ICTUS trial, which enrolled 1200 patients with an average age of 62 years, the invasive approach did not show superiority, yet it resulted in significantly lower rates of re-hospitalization [44].

However, it should be noted that these study findings may be influenced by biases in patient selection. A meta-analysis of these trials demonstrated a decrease in the risk of reinfarction and rehospitalizations, although a reduction in all-cause mortality is not achieved through a routine invasive strategy. However, this approach was associated with an increase in periprocedural complications and a heightened risk of bleeding [45].

Therefore, even older patients benefit from invasive treatment in terms of mortality reduction, although this benefit is accompanied by an increased risk of bleeding [46].

Conversely, the Italian Elderly ACS, RINCAL, and MOSCOW-FRAIL studies found no disparities between routine invasive treatment and conservative management in elderly patients with NSTEMI. Comorbidities and frailty strongly influence the outcome of elderly patients with ACS [47,48,49]. Indeed, among NSTEMI patients, the composite outcome of AMI, urgent repeat revascularization, stroke, significant bleeding, and all-cause mortality at one year occurred more frequently in frail patients [47,48,49].

In a study including NSTEMI patients aged ≥ 80 years, the primary outcome (composite of AMI, urgent revascularization, stroke, or death) was significantly reduced in patients assigned to the invasive strategy compared with the conservative one). Notably, there were no significant differences in bleeding complications between the two groups, and these favorable outcomes were sustained over a period of 5 years [50].

Notably, the efficacy of the invasive strategy with increasing age is more debated, particularly in those aged more than 90 years [50].

A meta-analysis on 1479 NSTEMI participants found no significant reduction in the risk of a composite outcome of death from any cause or AMI within 1 year with routine invasive management compared to conservative care in older patients. Nevertheless, lower rates of AMI and urgent revascularization have been reported in patients undergoing invasive options [51].

Another study involving 313 adults with NSTEMI, with a median age of 82 years, comparing the risks and benefits of an early interventional strategy, revealed a positive association between baseline troponin levels and the reduction in the primary endpoint, a composite of mortality, AMI, stroke, and hospitalizations within one year. These results suggest that troponin levels at admission may be considered when selecting the most appropriate treatment strategy [51].

Moreover, a small study on NSTEMI patients aged more than 80 years, no statistically significant difference in MACCE outcomes were found comparing interventional and conservative strategy at the 12-month follow-up. However, these results may be attributable to the limited sample size [52].

Importantly, among patients with NSTEMI, AMI, urgent repeat revascularization, stroke, significant bleeding, and all-cause mortality at one year seem to be more prevalent among frail patients compared to non-frail ones [53].

In a study involving 202 patients aged over 75 years with ACS, frailty was identified as an independent predictor of a composite outcome comprising death or major cardiovascular events in elderly patients with high-risk ACS. Frail patients exhibited a higher-risk profile and consistently experienced worse outcomes, regardless of the treatment administered [54].

According to the findings of the LONGEVO-SCA registry, which included 531 patients aged over 80 years with NSTEMI (27.3% identified as frail), the effectiveness of invasive treatment was found to be diminished by the level of frailty. This led to the conclusion that as frailty increases, the benefit derived from invasive therapy decreases [55].

Among elderly Spanish ACS patients ≥ 80 years, mortality and readmission were correlated with the severity of chronic kidney disease [55].

Elderly patients often have multivessel disease (MVD), and complete revascularization has been supported by numerous studies. While a reduction in hospital stays, risk of contrast-induced nephropathy (CIN), and other periprocedural complications can be lowered by treating only the culprit vessel, complete revascularization should be preferred [56].

The benefits of complete revascularization compared to culprit-only PCI have been demonstrated by both the COMPLETE [57] and PRAMI [58] trials for patients with acute myocardial infarction (AMI) and MVD. However, there is limited information on whether these benefits extend to the older adult population. These individuals often face higher CV risks and deal with various geriatric conditions. Therefore, elective interventions for non-culprit lesions in older patients are often postponed for fear of adverse events and the complexity of their MVD. According to the results of a FIRE [59] study on 1445 STEMI and NSTEMI patients with MVD, aged > 75 and a median age of 80 years, comparing complete PCI to a culprit-only PCI, physiology-guided complete revascularization leads to better outcomes compared to revascularization of only the culprit lesion. This improvement in outcomes is consistent even in patients with a high bleeding risk. The rates of acute kidney injury, stroke, or major bleeding were comparable between the two treatment groups [60].

In another study comparing the interventional strategy to the conservative one, NSTEMI frail patients did not experiment with a decreased length of hospitalization when invasively treated, with a greater incidence of bleeding [61].

A clinical randomized trial involving 1518 NSTEMI patients over the age of 75, which compared conservative and invasive approaches without excluding those with frailty and multiple comorbidities, demonstrated no significant reduction in CV mortality or nonfatal AMI in patients treated with an invasive strategy compared to those managed conservatively over a median follow-up period of 4.1 years [52].

Therefore, in cases of MVD, performing complete revascularization should be preferred to performing PCI exclusively on the culprit artery. However, it remains unclear whether these study findings can be extrapolated to frail patients.

In this sense, conducting a comprehensive assessment of frailty, including a multi-integrated assessment of physical condition, cognitive abilities, and overall functional status, assumes a pivotal importance (3) [62] A careful approach to patient care, considering factors like frailty, comorbidities, function, cognitive impairment, and polypharmacy—each of which increases the risks associated with invasive procedures—has been suggested to improve outcomes [22].

Frailty should be assessed using specific tools in patients undergoing procedures helping to discriminate the most suitable candidates to an invasive approach.

## 5. Frailty

**Figure 3:**
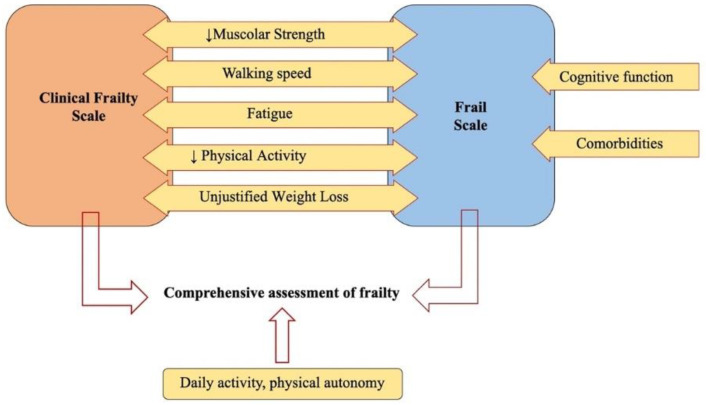
Geriatric assessment: Geriatric syndromes increase in elderly in whom multimorbidity, polypharmacy frailty, cognitive impairment, and physical decline often occur, leading to a higher risk of adverse outcomes. ↓: reduction of.

Identifying, assessing, and managing frail elderly patients is at the core of geriatric medicine. Frailty is defined as a state of increased vulnerability to stressors due to diminished physiological reserves, which leads to adverse outcomes following clinical events or cardiovascular interventions. While frailty precedes disability, it is distinct from it, though they may overlap. Despite the absence of a unanimous consensus on the optimal method for measuring frailty, two widely accepted models are the frailty phenotype and the deficit accumulation model. The frailty phenotype describes frailty as a clinical syndrome marked by three or more of the following: muscle weakness, slow gait, physical inactivity, weight loss, and fatigue. In contrast, the deficit accumulation model quantifies frailty through a frailty index, which reflects the proportion of age-related health deficits. When frailty is detected through screening or is evident, a comprehensive evaluation of specific deficits, or a full geriatric assessment, is recommended [63] (Figure 3). In these patients, it is crucial to assess frailty, disability, cognitive deficits, and comorbidities, such as renal disease, before any procedure to improve quality of life. Prolonged hospital stays can worsen quality of life, contributing to cognitive decline, malnutrition, and eventually sarcopenia. A comprehensive geriatric assessment should include evaluations of comorbidities (using tools like CIRCS and the Charlson Comorbidity Index, CCI), polypharmacy and the risk of drug interactions or non-adherence, autonomy (through ADL and IADL scales), functional status (with the SPMSQ and Mini-Mental State Examination), nutritional status, and sarcopenia (assessed with the Short Physical Performance Battery, SPPB). This detailed assessment is crucial in determining which patients will truly benefit from interventional procedures. The Mini Nutritional Assessment (MNA) can be employed as a screening tool for malnutrition [64]. However, body mass index (BMI) can also serve as a useful indicator for assessing nutritional status in elderly patients. Additionally, hypoalbuminemia (albumin levels < 30 g/L) may indicate severe malnutrition [65]. Frailty often shares a low-grade inflammatory state, known as “inflammaging,” with cardiovascular diseases. Geriatric syndromes can significantly affect clinical presentation, disease progression, decision making about interventions, and outcomes. In particular, frailty is a key predictor of adverse events, making its assessment essential in deciding whether to proceed with coronary angiography. Elderly patients with ACS present numerous challenges, and implementing a comprehensive geriatric assessment in real-world settings is often difficult. There is a need for a standardized and uniform frailty assessment tool, specifically tailored for hospitalized patients. Telemedicine could play a valuable role, both in diagnosing ACS—since symptoms are not always specific—and in providing regular follow-up to monitor patient stability [63]. A patient-centered care approached has the potential to reduce hospitalizations by improving adherence to medical therapies. Moreover, caregiver involvement is crucial in offering additional support to the patient. In the future, a more comprehensive management of all geriatric syndromes could lead to improved outcomes for these patients [63].

### 5.1. Medical Therapy and Prognosis in Acute Coronary Syndromes (ACS) among Older Adults

In elderly patients, comorbidities and multimorbidity often lead to polypharmacy, increasing the risk of numerous drug interactions [66]. Aging also brings about significant changes in pharmacokinetics and pharmacodynamics [67]. Serum proteins, particularly albumin, tend to decrease in advanced age. Since the concentration of unbound drugs largely determines pharmacodynamic activity, drugs with high protein binding—such as warfarin, most statins (except pravastatin), and amiodarone—may have enhanced effects in older adults [62]. Additionally, decreased levels of P-glycoprotein and cytochrome P450 enzymes contribute to increased concentrations of statins, beta-blockers, and warfarin [62]. Liver mass declines by approximately 10%–15% per decade in women, accompanied by a 60% reduction in hepatic blood flow in both sexes [29]. Despite these changes, hepatic metabolism remains substantially unaffected by age [28,29,68]. Renal function also declines with aging, more pronouncedly in women. However, the rate of decline is highly variable, and in many cases, no significant age-related reduction in renal function is observed. Evaluating renal function using serum creatinine levels may be confounded by reduced muscle mass in older women, potentially leading to an underestimation of renal impairment [29]. Pharmacodynamics in older adults is influenced by alterations in receptor expression and affinity, changes in second messenger systems, and modifications in cellular and homeostatic regulatory mechanisms [69]. The effects of similar drug concentrations at the site of action may vary due to changes in drug–receptor interactions, post-receptor events, or homeostatic responses. In frail individuals, these effects may also be influenced by underlying organ damage [70]. Nonetheless, aging itself is associated with common adverse drug reactions (ADRs), such as hypokalemia induced by intravenous furosemide or delirium resulting from antibiotics, antiarrhythmics, or digoxin [71]. ADRs in older adults often arise from variations in renal tubular secretion, intracellular free calcium levels, blood–brain barrier permeability, and postsynaptic choline content, among other factors [71]. Additionally, reduced peripheral alpha-adrenergic secretion and receptor expression with advanced age leads to a higher incidence of orthostatic hypotension from calcium antagonists or alpha-adrenergic blockers. Although older patients are generally less sensitive to the cardiac effects of beta-blockers due to reduced beta-receptor expression, orthostatic hypotension remains a frequent ADR with these agents [62]. Older women may be at increased risk of drug toxicity, primarily due to elevated drug serum levels [28,29,68]. However, specific studies are needed to better understand the challenges of drug therapy in this population. A meta-analysis involving 186,854 participants, of whom 14,483 (8%) were over 75 years old at the time of randomization, with a follow-up duration of 4.9 years, demonstrated that LDL reduction effectively lowers cardiovascular events. Statins appear beneficial for secondary prevention in the elderly, although their role in primary prevention remains controversial [72]. Evidence supports the use of statins regardless of age, particularly for secondary prevention when administered at moderate to high intensity. However, their use is not always consistent due to the presence of comorbidities and cognitive deficits, even though no direct correlation between statin uses and cognitive decline has been established. Additionally, underprescribing statins is often linked to concerns about side effects such as myalgias, hepatotoxicity, and rhabdomyolysis [73]. Ezetimibe and PCSK9 (proprotein convertase subtilisin/kexin type 9) inhibitors have also shown beneficial effects in preventing ischemic events [73]. Beta-blockers are effective for anti-ischemic treatment and preventing atrial fibrillation. However, their use in elderly patients requires individualized consideration due to potential bradycardic and negative inotropic effects. The long-term benefits of beta-blockers in this population have not been conclusively demonstrated. A low dosage of lisinopril or valsartan may be beneficial for elderly patients with heart failure, though careful attention should be given to renal insufficiency and hyperkaliemia. The decision to use ACE inhibitors or ARNI (angiotensin receptor-neprilysin inhibitors) in elderly patients should consider renal function, the risk of hyperkalemia, and potential arterial hypotension, as well as long-term benefits and life expectancy. According to the latest guidelines, lifestyle modifications, including dietary changes and physical activity, are recommended [74,75,76,77]. However, these interventions are only feasible for elderly patients who are physically fit. The PARADISE-MI study, which compared ARNI treatment with ACE inhibitors in patient’s post-myocardial infarction with left-ventricular dysfunction, found that sacubitril-valsartan significantly reduced the incidence of mortality from cardiovascular causes and heart failure. For patients over 75 years of age, high-intensity statin therapy should be initiated to achieve an LDL reduction of 50% [59]. Medical therapy for ACS is generally similar across age groups; however, it is crucial to account for physiological variations in older patients and adjust dosages accordingly.

### 5.2. Antithrombotic Therapy in Elderly ACS Patients

Antithrombotic therapy (ATT) for older patients (defined as >75 years of age) who present with an ACS follows the same general indications adopted for younger age groups [78]. However, several considerations, linked specifically to age, influence the choice of one agent over another, its dosing, and the duration of treatment [79] (Table 1). Age enhances the bleeding side effects of ATT [80], as well as the ischemic risk associated with undertreatment; therefore, this therapy should be undertaken carefully, particularly in the elderly, to avoid potentially fatal or disabling bleeding, on the one hand, or ischemic events on the other.

In the absence of a clear contraindication (such as recent major surgery, active bleeding, or severe liver dysfunction), and regardless of age, the general ATT approach to all types of ACS (STEMI, NSTEMI, and UA) includes initial parenteral anticoagulation and (mainly oral) dual antiplatelet therapy (DAPT) consisting of aspirin and a P2Y12-receptor inhibitor. Anticoagulation is extended up to the time of percutaneous coronary intervention (PCI) or hospital discharge [78]. Oral DAPT is extended up to 12 months after the index event (and even beyond, in high-ischemic-risk patients), followed by antiplatelet monotherapy [78,79]. The latter typically consists of aspirin 100 mg daily, but clopidogrel 75 mg daily is a valid alternative [78,79]. A recent longitudinal study of 7966 European patients discharged after an ACS and followed for one year has reported substantial undertreatment with antiplatelet agents at 30 days and one year, attributable at least in part to older age, comorbidities (including concomitant indications for long-term anticoagulation [81,82,83]), and lack of revascularization [84].

Aspirin is administered as soon as the diagnosis of ACS is confirmed as an oral 150–300 mg loading dose (or 75–250 mg intravenously if oral intake is impossible), followed by 100 mg daily [78,79]. It is debated, especially among patients not undergoing primary PCI, whether a P2Y12-receptor inhibitor on top of aspirin should be administered before coronary angiography (on the rationale of earlier antiplatelet effects) or deferred until the time of PCI (thereby reducing bleeding risks) [78,85].

Oral prasugrel (60 mg load followed by 10 mg daily, for ACS patients in whom PCI is planned) and oral ticagrelor (180 mg load followed by 90 mg twice daily, for all ACS patients) have each shown a significant reduction in ischemic risk during the first year after ACS, when compared to oral clopidogrel (300–600 mg load followed by 75 mg daily), but at the cost of significantly increased major bleeding for prasugrel (hazard ratio 1.32) [86] and fatal intracranial hemorrhages for ticagrelor [87].

For patients > 75 years, prasugrel is generally discouraged and, if used, the appropriate maintenance dose is 5 instead of 10 mg daily [78,79]. For elderly ACS patients, especially those at enhanced risk of bleeding or not at high ischemic risk, choosing clopidogrel as the P2Y12-receptor inhibitor and shortening DAPT duration to less than 12 months are reasonable options [78,79].

When PCI is planned (e.g., for STEMI or other ACS associated with raised circulating troponin levels), anticoagulation with i.v. unfractionated heparin (bolus < 5000 IU, followed by infusion < 1000 IU per hour, adjusted to activated partial thromboplastin time of 1–5–2.5 times control) is preferred over s.c. Enoxaparin (1 mg/kg twice daily) or i.v. Bivalirudin (bolus plus infusion; mainly for STEMI), with a class of recommendation I versus IIa [78]. If PCI instead is not planned, s.c. fondaparinux 2.5 mg daily is preferred over s.c. enoxaparin, given reduced major bleeding and mortality [88], but with increased thrombosis and other complications during catheterization [89]. Impaired creatinine clearance contraindicates bivalirudin if <30 mL/min, fondaparinux if <20, and enoxaparin if <15 [78,79]. Moreover, the enoxaparin dose is reduced by 25% for age > 75 years and halved if creatinine clearance is 15–29 mL/min [78,79].

Regardless of age, patients with acute STEMI presenting within 12 h of symptom onset should undergo PCI within 90 min of first medical attention (primary PCI) [39,78]. If primary PCI is not achievable within 2 h of first medical contact, STEMI patients should receive systemic fibrinolysis (tenecteplase, alteplase or reteplase, provided there are no absolute contraindications), followed by PCI within 24 h [39,78,90]. For STEMI patients > 75 years, the dose of tenecteplase (a genetically engineered triple variant of tissue-type plasminogen activator administered as a single i.v. bolus) is reduced to one-half [79,90]. Furthermore, for patients older than 75 years, the clopidogrel loading dose accompanying fibrinolysis is 75 instead of 300 mg [78,79]. Anticoagulation is usually co-administered with fibrinolysis, although randomized, placebo-controlled trials are lacking, especially among older patients. The general recommendation is to extend anticoagulation until revascularization (if performed) or up to 8 days of hospitalization [78].

The duration of DAPT is calibrated according to the patient’s bleeding risk. The latter is estimated using validated tools such as the 5-item PRECISE-DAPT calculator (age, hemoglobin, white cell count, creatinine clearance, prior spontaneous bleeding) or the Academic Research Consortium High Bleeding Risk (ARC HBR) definition (based on one major or two minor risk criteria, with age > 75 years representing a minor criterion) [91,92]. A shortened DAPT duration after PCI (equal to or less than 6 months) should be considered for PRECISE-DAPT scores > 25 or ARC-HBR [78,79,91,92]. A DAPT duration as short as one month for elderly PCI patients, even after complex procedures, is supported by the recent MASTER-DAPT trial conducted in HBR acute and chronic coronary syndrome patients followed for 11 months; in that trial, age > 75 years was a sufficient qualifying HBR criterion [93].

Bleeding risk needs to be balanced against ischemic risk. The validated DAPT prediction rule, based on the DAPT trial, is unique because it incorporates in a single score the prediction of reduced ischemic event rates and increased bleeding rates associated with DAPT extension beyond 12 months from PCI [94]. Of note, ticagrelor-based DAPT was not investigated in the DAPT trial. Scores >2 favor DAPT extension, and <2 suggest bleeding-related harm with prolonged DAPT. According to this rule, age > 65 years (score −1) or >75 years (score −2) convey distinct, enhanced bleeding risk, discouraging DAPT prolongation in elderly ACS-PCI patients unless multiple ischemic enhancers are present. The latter include current smoking, diabetes, prior MI/PCI, or stent type (each 1 point), and vein graft PCI or heart failure with reduced ejection fraction (each 2 points) [94].

### 5.3. Cardiac Rehabilitation

Cardiac rehabilitation (CR) programs have been suggested for patients undergoing percutaneous or surgical revascularization procedures [95].

A multidisciplinary team (MDT), including geriatricians, may determine the suitability of rehabilitation treatment, aiming to optimize the patient’s recovery and overall outcomes following treatment for their condition [96].

Indeed, identifying frailty during hospitalization is crucial for effective treatment, as addressing it can lead to improved prognoses. The CR for frail patients should be progressive and tailored to individual needs, incorporating aerobic, resistance, and balance exercises. Elderly patients derive more significant benefits from CR, enhancing their quality of life [97,98].

An accurate clinical assessment and multidisciplinary geriatric evaluation can help identify the appropriate individualized rehabilitation program. For elderly patients, the goals are to enhance quality of life by restoring physical activity, improving balance to reduce the risk of falls, preventing sarcopenia, and promoting socialization [99].

To enhance adherence, hybrid rehabilitation programs designed for elderly patients, combining home-based and organized care settings, should be adopted. These programs should integrate psychosocial counselling to promote the maintenance of healthy lifestyles.

However, in Europe, adherence rates to CR programs are low due to various barriers, including physical conditions, the presence of comorbidities, cognitive impairment, transportation difficulties, and lack of social and emotional support [100].

## 6. Future Perspective

Age is a well-recognized risk factor for the development of ACS, with NSTEMI being the predominant subtype in adults aged over 75 years. Due to the underrepresentation of older patients in RCT, there is a lack of evidence in terms of a pharmacological and interventional approach for elderly individuals with ACS. Moreover, studies focusing on older patients with ACS undergoing an invasive approach are hindered by small sample sizes and the lack of a comprehensive evaluation of frailty, comorbidities, and cognitive function. This limitation reduces the practical value of the available data. The absence of strong evidence has been highlighted in international guidelines, which are often not specific for the elderly due to their complexity and heterogeneity, leading to a lack of specific evidence [101]. These factors highlight the significant differences observed among individuals of the same chronological age with heart disease, which include cardiovascular aging, the presence of cardiac pathology, comorbidities, disability, frailty, lifestyle, and socio-environmental factors. This gap complicates therapeutic decision making, as it requires balancing the expected clinical benefits with the potential risks of therapies and procedures [101]. Furthermore, clinical presentation in this population is multifaceted; therefore, frailty, comorbidities, cognitive function, and health-related quality of life should be crucial factors for clinical management decisions It is essential to identify patients who are neither “too healthy” nor “too ill” to benefit from medical interventions, while also recognizing those for whom care may be futile. Frailty, in particular, should not be considered a sufficient reason to withhold treatment from an elderly patient. Rather, its evaluation should guide personalized and appropriate care and a tailored approach taking into consideration specific characteristics of each patient should be advisable.

## 7. Conclusions

Establishing the optimal treatment strategy for elderly patients with ACS presents significant challenges. Older patients exhibit a higher susceptibility to complications such as stroke, renal dysfunction, respiratory impairment, and hemorrhagic complications. Consequently, accurately evaluating the presence and severity of non-cardiac comorbidities balancing the upsides and downsides of different therapeutic strategies is crucial.

Evaluating the patient’s cognitive and functional status holds paramount significance.

Frailty is becoming an important factor in assessing and guiding treatment decisions for older adults, particularly in the context of ACS considering the higher risk of adverse events and complications.

For older patients ACS, a personalized strategy is essential. Treatment decisions should take into account the patient’s functional and cognitive abilities, along with their overall quality of life, before opting for aggressive interventions.

## Figures and Tables

**Table 1:** Adjustments of antithrombotic doses according to age.

Antithrombotic Therapy	Young Patients	Elderly (Defined as >75 Years of Age)	
Aspirin	Oral 150–300 mg loading dose (or 75–250 mg intravenously if oral intake is impossible followed by 100 mg daily	The same dose	
Prasugrel	60 mg loading dose followed by 10 mg daily	Decrease dose to 5 mg/daily	Avoid prior stroke
Ticagrelor	180 mg load followed by 90 mg twice daily	The same dose	
Clopidogrel	300–600 mg load followed by 75 mg daily	The same dose	Decrease risk of bleeding
Clopidogrel		No loading dose with fibrinolysis	
Enoxaparin	1 mg/kg	Reduce 0.75 mg for age > 75 years, and half dose if creatinine clearance is 15–30 mL/min	
Tenecteplase	*Tenecteplase* single IV bolus based on weight	Half dose	
Fondaparinux	2.5 mg	Avoid if CrCl < 20 mL/min	
Bivaliridina	0.75 mg/Kg/ev	Avoid if CrCl < 30 mL/min. Reduce infusion to 1.4 mg/kg/h if CrCl 30–59 mL/min	
*UFH* IV dose adjusted to aPTT		No reduction	
Cangrelor 30 μg/kg bolus + immediate 4 μg/kg/min infusion for ≥2 h. Oral P2Y_12_ inhibitor load during (for ticagrelor or prasugrel) or at end (for clopidogrel) of infusion		The same dose	The same dose
Other drugs and and duration of DAPT		PPI in older Elective PCI DAPT for 1 month For SCA 6–12 months (depending on bleeding and ischaemic risk balance)	

## Data Availability

No new data were created or analyzed in this study.
